# 

**Published:** 1885-05

**Authors:** 


### PERSONALS.

Dr. E. Schmidt has been reëlected attending surgeon, and Professor J. E. Owens has been elected consulting surgeon of Michael Rees Hospital.

Drs. T. McDill, H. H. Frothingham, — — Hartman, F. W. Harkness, A. W. Caldwell, S. P. Black, E. Van Hook, and T. Davis have been appointed resident physicians to Cook County Hospital.

Drs. J. J. Alderson, N. H. Pierce and C. A. Foulks have been appointed resident physicians to St. Luke's Hospital.

Drs. E. S. Parker and L. E. Lawson have been appointed resident physicians to Mercy Hospital.

Drs. R. G. Collins and L. E. Frankenthal have been appointed resident physicians to Michael Rees Hospital.

Dr. F. Eggleston has been appointed resident physician to the U. S. Marine Hospital.

Dr. J. H. Hoelscher has been appointed resident physician to the Alexian Brothers Hospital.

Dr. L. L. McArthur has been appointed to the surgical department of the Dispensary of St. Luke's Hospital.

SUBSCRIPTION PRICE, $3.00 PER YEAR.

Whole Number, 305. MAY, 1885. Vol. L., Number 5.

INDEX MEDICUS

LIBRARY INDEXED S.G.O.

### THE CHICAGO MEDICAL JOURNAL AND EXAMINER

EDITORS:

S. J. JONES, M.D., LL.D.

W. W. JAGGARD, A.M, M.D. HAROLD N. MOYER, M.D.

---

COLLABORATORS.

CHICAGO:

PROFESSOR J. A. ALLEN, M. D., LL. D.
PROFESSOR E. ANDREWS, M. D., LL. D.
PROFESSOR W. H. BYFORD, A. M., M. D.
PROFESSOR O. C. DEWOLF, A. M., M. D.
PROFESSOR E. C. DUDLEY, A. B., M. D.
PROFESSOR J. H. ETHERIDGE, A. M., M. D.
PROFESSOR C. FENGER, M. D.
PROFESSOR J. N. HYDE, A. M., M. D.
PROFESSOR H. A. JOHNSON, M. D., LL. D.
CHARLES GILMAN SMITH, A. M., M.D.
J. F. TODD, M. D.

MILWAUKEE, WISCONSIN:
N. SENN, M, D.

WASHINGTON, D. C:
S. O. RICHEY, M. D.

UNITED STATES NAVY:
MEDICAL DIRECTOR J. M. BROWNE, M. D.

CHICAGO:

